# Human Bocavirus in Febrile Children, the Netherlands

**DOI:** 10.3201/eid1301.060819

**Published:** 2007-01

**Authors:** Miriam Monteny, Hubert G.M. Niesters, Henriëtte A. Moll, Marjolein Y. Berger

**Affiliations:** * Erasmus MC, Rotterdam, the Netherlands

**Keywords:** parvoviridae infections, human bocavirus, child, fever, communicable diseases, emerging, family practice, letter

**To the Editor**: Human bocavirus (HBoV) is a recently discovered virus of the family Parvoviridae, genus Bocavirus, which appears to cause widespread respiratory tract infections (RTI) in children. In selected groups of children with RTI, detection rates have varied from 2.8% to 11.3% (*1**–**9*). However, the exact prevalence and pathogenic effects of this virus remain to be established.

During a prospective cohort study to evaluate the prognosis of fever at a general practice after-hours service in Rotterdam, nasopharyngeal swabs were collected from febrile children and tested for respiratory viruses, including HBoV. We report the incidence and clinical features of HBoV infection in these children.

From June 1, 2005, through January 16, 2006, all children 3 months to 6 years of age whose parents contacted the after-hours service because of fever, as reported by parents and not further defined, were eligible for inclusion in the study. Children were excluded when the parents could not communicate in Dutch (n = 77) and if the child had already been included within the past 2 weeks (n = 11). A research nurse visited the child at home within 24 hours of inclusion. The child was physically examined, and a nasopharyngeal swab and a blood sample for C-reactive protein measurement were collected. The parents subsequently recorded the child's symptoms in a diary for 7 days. The Central Committee on Research Involving Human Subjects, the Netherlands, approved this study.

Nucleic acids were isolated on a MagnaPure isolation station (Roche Applied Science, Penzberg, Germany) and subsequently analyzed by real-time assays. Detection of HBoV was performed by using a primers set and a fluorescein amadite–labeled TaqMan probe directed against sequences of the NP1 gene. (Sequences are available from the corresponding author.) Testing was routinely done for the following viruses: influenza virus types A and B, parainfluenza virus types 1–4, respiratory syncytial virus (RSV) types A and B, adenovirus, coronavirus (OC43, 229E, and NL63), and rhinovirus.

Nasopharyngeal swabs were collected from 257 (81%) of 319 enrolled children. The overall virus detection rate was 52.9%; most frequently detected were adenovirus (11%), RSV-A (10.5%), parainfluenza virus type 1 (8.5%), and rhinovirus (8%). Five children were included twice; none of them was HBoV positive. The PCR for HBoV was positive in 4 children (1.6%), all boys. The characteristics of these children are shown in the Table.

**Table:** Characteristics of children with human bocavirus infection, the Netherlands*

Patient no.	Age, y	Month detected	Symptoms	Body temperature (°C)	CRP (mg/L)	Positive PCR (log copies/mL)
1	2.3	Nov 2005	Earache, rhinorrhea, cough, sore throat, abdominal pain, diarrhea, skin rash, dyspnea, increased breathing rate	36.2	9	Bocavirus (4.20)
2	1.9	Nov 2005	Rhinorrhea, cough, sore throat, vomiting, increased breathing rate	38.8	26	Rhinovirus (7.57), RSV B (5.51), bocavirus (2.88)
3	1.3	Dec 2005	Earache, rhinorrhea, cough, headache, vomiting, skin rash, increased breathing rate	38.6	26	Bocavirus (6.72), para-influenza virus 4 (4.11), adenovirus (2.00)
4	1.0	Jan 2006	Earache, rhinorrhea, cough, sore throat, abdominal pain, diarrhea, vomiting	37.7	88	Adenovirus (6.90), bocavirus (2.88)

*CRP, C-reactive protein; RSV, respiratory syncytial virus.

All 4 children reported rhinorrhea and cough. Patient 1 reported abdominal pain, diarrhea (more than twice daily, with mucus), dyspnea, and a skin rash, along with respiratory symptoms. All symptoms lasted for >1 week. At physical examination, the children were given a score by research nurses on whether they did not appear ill or appeared slightly ill based on standard criteria. Patient 1 had a skin rash and palpable cervical lymph nodes. Patient 4 had palpable lymph nodes and red tonsils.

Patient 1 was given amoxicillin for otitis media, and patient 4 received amoxicillin for tonsillitis, as diagnosed by the general practitioner on the basis of the patient's clinical symptoms, without bacteriologic confirmation. During a 1-week follow-up period, none of the patients sought further medical advice.

Our finding that HBoV may cause RTI is in accordance with the literature (*1**–**6*). Our findings support those of others in suggesting a role for HBoV in systemic infection, causing gastrointestinal symptoms and skin rash (*6**,**8*).

Our detection rate, in general practice, is lower than the rates reported from former studies of children with RTI (3%–10%) (*1**–**8*). Coinfection of HBoV and other viruses was found among 3 (75%) of 4 children (Table). In other studies, coinfection was found in 17.6%–55.6% (mainly adenovirus, RSV, and human metapneumovirus) (*1**,**2**,**5**,**7**–**9*). The other detected viruses could have caused the symptoms of patients 2–4. However, HBoV was the only detected virus in 1 child with respiratory symptoms, gastrointestinal symptoms, and rash, and therefore might be the pathogen. Considering the high amount of HBoV in patient 3, symptoms were likely caused by HBoV in this patient as well.

Our ability to analyze severity of disease in our study population was limited because all children had mild disease, and no child was hospitalized. However, all children reported a prolonged course of fever, >7 days or recurrent within 1 week. This finding is in contrast with the mean duration of fever of 2.6 days in the study of Arnold et al., which was based in a hospital setting (*6*). In our study, none of the HBoV-positive children received a diagnosis of bronchiolitis, pneumonia, or bronchitis, as in previous studies. None of the 4 children in our study was born preterm, compared with 19%–44% of HBoV-positive children in previous studies (*5**,**6*). None had a positive history for asthma or other underlying diseases, as did up to 50% of the children in previous studies (*1**,**6*).

In conclusion, HBoV was detected in nasopharyngeal swabs of 4 (1.6%) of 257 children <6 years of age who parents contacted a general practice after-hours service. Our results suggest that HBoV might cause mild disease with respiratory and gastrointestinal symptoms and skin rash. Further research, not restricted to susceptible or hospitalized patients, is needed to clarify the prevalence and pathogenicity of this new virus in the general population.
